# A New Perspective on Agitation in Alzheimer’s Disease: A Potential Paradigm Shift

**DOI:** 10.3390/ijms26073370

**Published:** 2025-04-04

**Authors:** John R. Ostergaard

**Affiliations:** Centre for Rare Diseases, Aarhus University Hospital, Palle Juul-Jensens Boulevard 99, DK-8200 Aarhus, Denmark; john.oestergaard@skejby.rm.dk; Tel.: +45-5124-4546

**Keywords:** Alzheimer’s disease, autonomic nervous system, heart rate variability, neurodegeneration, parasympathetic withdrawal, sympathetic overactivity, transcutaneous vagal stimulation, neuronal ceroid lipofuscinosis

## Abstract

Agitation is a common and difficult-to-manage neuropsychiatric syndrome in dementia. Recently, an association with the autonomous nervous system has been suggested. From the literature researched, however, only two studies investigating autonomic function concomitant to agitation situations appeared; one case series comprised two American veterans with vascular and Alzheimer’s dementia, respectively, and in a case series of patients with CLN3 (juvenile neuronal ceroid lipofuscinosis), this was found to be the most common neurodegenerative disease leading to dementia in childhood. In both case series, the measurement of the autonomic system disclosed a parasympathetic withdrawal and sympathetic hyperactivity in the temporal context with agitated behavior. If the time-wise-related autonomic imbalance shown previously can be demonstrated in a larger cohort of patients with Alzheimer’s disease, the use of transcutaneous vagal stimulation might be a potential paradigm shift in the treatment of agitation in Alzheimer’s disease.

## 1. Introduction

The main clinical manifestation of Alzheimer’s disease (AD), the most common form of adult dementia, is cognitive impairment. However, more than 75% of patients have also shown various behavioral and psychological manifestations, collectively known as neuropsychiatric symptoms [1], contributing to the complexity of medical and nursing care, as well as caregiver burden [2]. Both clinicians and family caregivers identify agitation and anger as the most concerning behavioral manifestation during the late stages of dementia; clinicians focus primarily on the safety risks that agitation can pose, while family caregivers become highly distressed with agitation, altering the personality of their relatives [3]. Generally, different neuropsychiatric symptoms are related to specific cognitive impairments. Hallucinations are associated with decreased visuospatial function, and delusions are associated with decreased executive function, reasoning ability, and conceptualization [2]. Depression is related to a decline in executive and memory function [2]. Mental symptoms and agitation seem to be associated with more cognitive domains and more rapid cognitive decline. Both are accompanied by impaired language and memory function, while agitation is also associated with decreased executive function, visuospatial function, and conceptualization [2]. Apathy is closely related to executive function, whereas abnormal motor behavior is associated with both executive and language impairment [2]. Anxiety is primarily associated with damage to the subcortical regions, of which the amygdala seems to play an important role in risk assessment and response, and the locus coeruleus plays an important role in the efferent response systems [2]. All symptoms adversely reduce the quality of life of dementia patients, their relatives, and caregivers and are anticipated to be associated with the more rapid progression of dementia and earlier death [4,5]. The almost universal existence of neuropsychiatric symptoms combined with their disabling effects on patients and caregivers is contrasted by the fact that few effective and safe preventive and treatment measures exist. This is mainly attributed to the lack of reliable and effective measurements of neuropsychiatric manifestations and because the pathological mechanisms, including their etiologic pathways and interactions, largely remain unclear.

Cross-sectional and longitudinal studies have indicated that different neuropsychiatric symptoms occur at different stages of AD, but mostly, the symptoms appear in the preclinical AD or mild cognitive impairment stages and develop progressively [6,7,8]. This most probably reflects the progression of dementia disease and, thus, also of cognitive impairment over time. Apathy, depression, anxiety, and irritability occur especially in the preclinical and amnestic mild cognitive impairment stages, whereas agitation and abnormal motor behavior are prevailing symptoms in the moderately and especially in the most severe stages of AD [2]. Additionally, in the later phases, delusions and hallucinations may cause confusion between reality and morbid fantasies [2].

## 2. Agitation

### 2.1. Clinical Aspects

Agitation, defined as a sustained, observed, or inferred evidence of emotional distress, is clinically often associated with aggression, both verbal (e.g., yelling, screaming, shouting) or physical (e.g., grabbing, pushing, hitting, or kicking objects or people, throwing objects, slamming doors, destroying property) characteristics, and excessive motor activity like pacing, rocking, gesturing, pointing fingers, or performing repetitious mannerisms [1]. It affects around 80% of nursing home residents with AD [9]. It has been hypothesized that agitation in AD individuals could be an expression of anxiety [10,11,12,13], implying that the former could replace the latter as dementia progresses and that anxiety early in the course of dementia could increase the risk of later developing agitation. Consistent with this hypothesis, anxiety typically emerges in the preclinical stages of AD [12] and has a lower prevalence in those with severe AD, whereas agitation increases in prevalence with disease progression and the worsening severity of cognitive impairment [13,14]. Using a prospective design, Liu and coworkers [15] have recently questioned the latter hypothesis, but they similarly found a positive linear relationship between incident anxiety and incident agitation over the study duration, thus substantiating that a high rate of individuals with anxiety in the earlier phases of AD is followed by an increased incidence of episodes of agitation in the later stages of AD.

### 2.2. Neural Substrate

The proposed neural substrates of agitation have been the loss of frontal–subcortical control and imbalance of affective and executive function networks, and have been linked to alterations of the dynamic balance between cholinergic and monoaminergic neurotransmission, the degeneration of the serotonergic nucleus of the dorsal raphe leading to a relative dopaminergic preservation, and the fact that the progressive loss of noradrenergic neurons in the locus coeruleus may cause the frontal upregulation of adrenergic receptors and impaired cortex activity [16,17,18].

### 2.3. Actual Treatment Proposals

Current clinical guidelines recommend nonpharmacological approaches as first-line treatment for agitation in AD, and so far, brexpiprazole is the only drug approved by the FDA for agitation associated with AD [19]. It is an atypical antipsychotic with the most favorable efficacy/risk profile. Its effect has been ascribed to its partial agonism of the serotonin 5-HT1A receptor and the dopamine D2 and D3 receptors. As in other atypical antipsychotics used in older patients with dementia, it carries a “boxed warning” for an increased risk of death. Other concerns include urinary tract infections, somnolence, insomnia, and cardiovascular events. No serious cerebrovascular adverse events have been reported [19]. Risperidone has been approved for the short-term treatment of severe agitation and aggression in the European Union, United Kingdom, Australia, and Canada, among other countries, but like other atypical antipsychotics (quetiapine and olanzapine), it shows overall modest efficacy [20], and they all have different risk profiles. Risperidone and olanzapine are linked to cardiovascular and extrapyramidal risks, while quetiapine is associated with sedation. Since none have a clear safety advantage regarding mortality [21] individualized, risk–benefit assessments are critical when prescribing these drugs, and they should be used at the lowest effective dose for the shortest duration, as chronic use increases mortality [20]. For further details and a thorough description of repurposed and newly developed drugs for agitation in Alzheimer’s diseases, see ref. [19].

## 3. Aggression

From a biological perspective, aggression is understood as a survival tool. It is considered the most serious and prevalent non-cognitive symptom experienced in patients with dementia [22,23] and is defined as behavior that is intended to harm another individual who does not wish to be harmed [24]. In the present context, the definition requires evidence of emotional distress and requires one of the three observable types of behavior (excessive motor activity, verbal aggression, or physical aggression) [25]. Aggression is a major antecedent to institutionalization, increased costs, and caregiver burden, and leads to an overall poor prognosis [24]. It is most prevalent in the last stages of dementia [14]; verbally aggressive behaviors are associated with the female gender [26], whereas men display more physical aggression [26]. Once aggression is identified, possible causes (unmet needs) or triggers should be considered. They include constipation, pain, hunger, thirst, sleep disorders, environmental factors (light, noise, crowding), or frustration due to a feeling of insufficient assistance with the basic activities of daily living [27]. Aggression can be divided into impulsive (reactive) aggression and planned aggression; the latter is planned in advance and often associated with a reduced degree of empathy and with an intention to achieve personal benefit. The aggression occurring in dementia has an impulsive characteristic and is the result of an affective reaction to a provocation, where the person with dementia cannot resist sudden aggressive instincts triggered by an intense emotion of anger [28]. Anger is one of mankind’s three innate emotions [29,30] and occurs along with an increased activity of the autonomic nervous system [28]. This emphasizes that our innate, primary, and inextinguishable emotions (anger, fear, and joy) are not just “feelings” or mental states but are accompanied by physiological and behavioral changes that are expressed at three interrelated levels: the mental or psychological level, the physiological level, and the behavioral level [28]; these represent the three functional layers of the brain: the cerebral cortex, the reptilian brain, and the limbic system, respectively.

In the literature, there are incongruent data on how to distinguish aggression and agitation, and often, the two are combined into a single measure of agitation/aggression [22]. Aggression may also be labeled as a subgroup of agitation that explicitly focuses on physical and verbal attacks [31]. Using the same context, agitation is then defined as verbal non-aggression and agitation, which, by itself, can escalate into aggressive behaviors [31].

## 4. Emotions and Cognition

It is generally accepted that emotional and cognitive processes cannot be dissociated and that cognitive apprehension is critically involved in emotional experiences as well as in coping strategies [30]. In cases of childhood dementia [32], the clinical expression certainly also depends on the child’s age when the neurodegenerative process starts, how fast it progresses, and in which order it occurs [33,34].

Cognitive functions include perception, attention, memory, language, and related executive processes that include problem-solving, decision-making (including response selection), and planning. Executive functions are often effortful, attention-demanding, and require self-monitoring. These functions and characteristics are severely challenged and even largely absent in the later stages of dementia (stages 6–7 of the primary degenerative dementia scale of Reisberg and coworkers [35]; i.e., during the stages when the episodes of anger and aggressive agitation in AD occur). At this level, the affected person needs help with most daily tasks and can no longer remember what just happened, daily routines, and planned events, including those of great personal significance, and their cognitive abilities correspond to the so-called preoperational developmental phase where in the current context, the affected person, can imitate, but does not use real cognitive operations [36]. This means they do not have the cognitive ability to encounter a normal anxiety response representing a state of heightened arousal and general feelings of averseness *in the absence of an immediate threat* [37]. On the other hand, at these late and severe stages of dementia, anxiety is replaced by the developmental natural fear response that occurs in a person with an intellectual development corresponding to a child 1–2 years of age when they meet *a definite and known threat* [30,34,38,39,40]. Since both the emotions of anxiety and fear are related to threats, albeit with differences in the dimension of the threat’s imminence, the developmental distinction between anxiety and fear may seem artificial and striking from a clinical point of view. However, the applied developmental distinction between anxiety and fear is substantiated by physiological, autonomic cardiovascular measures, which, although not consistent, mostly reveal that fear is accompanied by a decrease in total peripheral resistance, while anxiety is accompanied by an increased total peripheral resistance suggesting that they altogether may also be based on two distinct behavioral systems [41], although not necessarily different neurophysiological systems.

Our emotional states are adaptive and promote avoidance to protect us from potential dangers, and current evidence from clinical and preclinical research suggests that activation of our “threat” system arises from a set of highly interconnected neural circuits, of which the amygdala, ventral hippocampus, and medial prefrontal cortex are key nodes [30,39,40]. The efferent pathways of the circuit are mediated through autonomic, neuroendocrine, and skeletal–motor responses. The hypothalamic–pituitary–adrenocortical axis triggers or facilitates endocrine (primarily catecholamines) and neuropeptide release, whereas the regulatory control of the skeletal muscle response is more complex, depending on whether subtle movements involving a few muscle groups of the facial muscles are essential or whether freezing of the body or escape and fight is required [41,42]. Autonomic activation is the earliest physiological response observed and is produced by the sympathetic and parasympathetic neural systems acting antagonistically to maintain the equilibrium of vital functions and provide specific responses that accompany and adjust in relation to physical efforts and mental activities [43].

## 5. The Autonomic Nervous System in the Elderly and in Alzheimer’s Disease

By sensing changes in biomechanical, biochemical, and thermal signals, the action of the autonomic nervous system adjusts organ function to ensure that these measures are kept within stable ranges that are necessary for the homeostatic integrity of the body. Autonomic control also supports the complexity of organism-level behaviors that are necessary for survival and reproduction. Here, autonomic responses do more than just facilitate motor action and recovery states: autonomic nervous control is integrated with affective, motivational, and cognitive processes. These, in turn, engender distinct patterns of autonomic and behavioral responses, which reflect strategies that ensure stability through changes that are generally normative and adaptive. If a threat or a deviation from the normal range of physiological functioning (triggered by changes in the external or internal environment) is signaled, this will trigger a concerted set of neurophysiological and psychological responses to ensure that the internal milieu is kept constant (for review, see ref. [44]). The sympathetic and the parasympathetic branches of the autonomic nervous system have opposing roles. The sympathetic system helps the body address challenges. The parasympathetic system helps the body relax and save energy when resting. The parasympathetic system also helps with nonurgent but necessary tasks, such as removing metabolic waste and digesting food. Aging shifts autonomic activity: through early and mid-adulthood, sympathetic and noradrenergic activity increases during resting states, while parasympathetic and vagal activity declines, and parasympathetic vagal nerve activity is about 80% lower in adults aged 60 compared to those aged 20 years old. These two autonomic branches have opposite effects on many different physiological functions. For instance, the sympathetic system increases heart rate and glucose levels, whereas the parasympathetic system decreases heart rate and glucose levels. High parasympathetic activity when resting indicates good health, while high sympathetic activity when resting is associated with stress and disease. But being able to activate the sympathetic system when challenged indicates optimal functioning. The activation of the sympathetic system produces increases in blood pressure, heart rate, sweating, and pupil dilation, making us ready for freeze, flight, or fight. Depending on the intensity and persistence of the normative stressor(s), adaptive sympathetic and parasympathetic effects take place. However, when the central sympathetic/parasympathetic balance is in some way disrupted, a disharmonious and possibly exaggerated response occurs. Aging by itself disrupts the dynamic balance and the circadian oscillations of the sympathetic and parasympathetic systems. In younger adults, sympathetic activity is higher during the day than the night, while parasympathetic activity is lower during the day than the night. Additionally, in the elderly, parasympathetic activity is lower, while at the same time, older individuals have higher sympathetic activity than younger adults.

### Autonomic Nervous System and Agitation

Among various autonomic function assessment techniques, heart rate variability (HRV) analysis stands out due to its non-invasiveness and patient-friendly nature and offers a comprehensive reflection of an individual’s autonomic functional status [45]. Using this technique, prospective comparative cohort studies have shown that lower parasympathetic activity and higher sympathetic-vagal imbalance in middle-aged individuals are associated with increased dementia risk [46,47] and that individuals with Alzheimer’s disease have a significant sympathetic predominance when compared to controls [48,49]. Additionally, an increased agitation risk in Alzheimer’s disease has been linked to age-related HRV changes [46]. On the other hand, and opposite to their own prediction, Liu and coworkers [10] have recently reported that compared to non-agitated Alzheimer’s disease individuals, agitated individuals showed a reduced overall longitudinal decline in HRV when measured over 22–26 years of follow-up, and further research is needed on the potential for HRV indices to be a marker of agitation propensity in Alzheimer’s disease. A major step towards clarifying a possible link between autonomic nervous system dysfunction and agitation is “on-line” bedside studies of autonomic function before, during, and following periods of agitation. In the literature, however, only one single clinical observational study exists measuring autonomic function in adult dementia concomitant to an agitation situation, and that is in two American war veterans with vascular and Alzheimer’s dementia, respectively [50]. In both cases, behavior marking an abrupt transition from a calm to an agitated, angry state was witnessed, slightly separating these episodes from the more common scenario of uncooperative behavior escalating into combative behavior [9]; i.e., their actual combativeness implies a “contempt for death and a willingness to fight” [37]. The agitated episodes of the war veterans seem similar to the natural developmental but exaggerated fear-response that occurs in adolescent patients with CLN3 disease, which is the most common cause of neurodegenerative diseases leading to childhood dementia [32,51], when either the adolescent is separated from their parents, exposed to loud, unpleasant sounds, or is lifted against gravity [34,52,53]. Noteworthy, in both the war veterans and the adolescent CLN3 patients, a significant, transient, parasympathetic reduction leading to a transient sympathetic overactivity occurred in the temporal context with episodes of agitation [50,53]. In the war veterans, autonomous activity was measured as a dysautonomic response consisting of parasympathetic withdrawal (increased heart rate) followed 30 s later by sympathetic activation (increased skin conductance) [50]; in the CLN3 adolescents, autonomic activity was evaluated by bedside HRV investigations showing a shortly preceding, and during the attack ongoing, withdrawal of parasympathetic activity, leading to a transient sympathetic overactivity [53].

A person’s fear/threat level is activated due to an interaction between genetic and environmental factors, and a specific psychological vulnerability focusing on particular events or circumstances during earlier life is important [54]. In this context, it is reasonable to suggest that the “battle ready” combativeness observed in the two veterans, in Reisberg stages 6–7, can be traced back to incidents that took place way back when they were young and in circumstances that have contributed to shaping their conceptual and environmental awareness at a vulnerable time in their lives, i.e., being at war. Similarly, the agitated fearful reaction in the adolescent CLN3 patients in Reisberg stages 6–7 reflects the pure, simple, and basic natural fear reaction that normally evolves in the first 1–2 years of life in children. They have not yet been influenced by later life’s violent fear- and anger-inducing experiences and, therefore, express a developmentally natural fear reaction when exposed to threats during stages of their dementia condition where their disease has reduced their cognitive abilities to the equivalent of a child at around 1 year of age [34].

## 6. Discussion

Although the neurodegenerative processes in Alzheimer’s disease and CLN3 disease both affect almost all the structures relating to central autonomic network alterations and the highly interconnected neural circuits of natural emotional and behavioral expressions, such functional and structural changes may be regarded as two facets of the same process. Additionally, in the elderly, the use of poly-medication, which may interfere with autonomic functions, often occurs. Taking these factors into consideration, it is difficult unambiguously to establish whether autonomic dysfunction in dementia is exclusively a consequence of the underlying pathology of this disease. However, the time-related connection between a threat-inducing event and the transient sympathetic hyperactivity documented in both the CLN3 patients and the war veterans [50,53] is a significant indication that there most probably is a causal relationship. Furthermore, patients with AD have higher cerebrospinal fluid levels of noradrenaline than controls, which is consistent with the possibility that Alzheimer’s disease-related pathology triggers noradrenergic hyperactivity [55]. On the other hand, certain comorbidities of normal aging are also associated with health issues that increase sympathetic activity; for instance, these include sleep apnea, excess weight increases, and declining function in organs such as the kidney or heart [55].

Increasing evidence suggests that disease pathogenesis leading to dementia, including Alzheimer’s disease (for review, see ref. [56]) and CLN diseases [51], is not restricted to the neuronal compartment but also includes interactions with immunological and inflammation mechanisms and glial cells. In the present context, it is therefore also important to consider the interactions between the immune and inflammatory systems and the autonomic nervous system, and indeed, by trying to keep a homeostatic balance between pro-inflammatory and anti-inflammatory responses, the autonomic nervous system has a great impact on the inflammatory processes and immune responses [57]. Sympathetic pathways mainly interfere in the earlier stages of the inflammatory process, while the parasympathetic nervous system is more important to regulate innate immune responses and cytokine functional effects in chronic processes [58,59,60], but we still need comprehensively to explore and understand the complex interplay between dementia, behavioral, and psychological mechanisms and autonomic, endocrine, immunological and inflammatory processes.

### A Paradigm Shift

If the temporally agitation-related autonomic imbalance demonstrated in CLN3 adolescents and adult war veterans proves to be reproducible in a larger cohort of patients with AD and/or other types of dementia, it has the potential to be a paradigm shift in how we understand and prevent and treat agitation in advanced stages of dementia, including Alzheimer’s disease.

Theoretically, the stimulation of the vagal nerve might be able to restore the sympathetic-parasympathetic imbalance [61]. Invasive cervical vagal nerve stimulation (iVNS) has been used for >50 years and is approved for the treatment of severe epilepsy, depression, and stroke rehabilitation [62]. Additionally, improved cognitive performance according to Alzheimer’s Disease Assessment Scale–Cognitive Subscale (ADAS-cog) and Mini-Mental State Examination (MMSE) scores has been reported in patients with Alzheimer’s disease following one year of iVNS treatment [63]. However, possible adverse events, including invasiveness, restrict iVNS use for research purposes. The transcutaneous stimulation (tVNS) of the cymba conchae of the left auricular branch (taVNS) is considered an effective non-invasive alternative [62,64]. This method has been used in patients with epilepsy, depression, or anxiety disorders [64]. Additionally, in healthy adults, daily taVNS for 2 weeks shifted a high sympathetic dominance toward parasympathetic dominance, improving autonomic balance in some individuals [65]. Side effects were minor and mainly included skin reddening and irritation [66]. The transcutaneous vagal stimulation is administered via two modified dot-like electrodes placed near the auricular branch of the vagal nerve in the cymba conchae of the left ear, which comprises afferent fibers connecting it to the main branch of the vagal nerve [67]. Through the electrodes placed on the skin over the cymba conchae, the left auricular nerve branch transmits the impulse received from the taVNS device to vagal nuclei connections to the spinal nucleus of the trigeminal nerve in the spinal cord and to the nucleus of the solitary tract, from which it can modulate activity in widespread subcortical and cortical areas of the brain, including the hippocampus, amygdala, locus coeruleus, and medial prefrontal cortex. These regions are all important nodes of our emotional regulation network associated with the appropriate interpretation of emotions and emotional regulation strategies [68,69,70].

The poststimulation latency of vagal somatosensory-evoked potentials is longer in older adults and greater in older adults with mild cognitive impairment or Alzheimer’s disease than in those with normal cognition [71,72]. Because age-related effects are seen on vagal somatosensory-evoked potential latencies but not on amplitudes, these results suggest age-related demyelination instead of the loss of neurons or fibers [71], and although the structure and function of different nerves have not been typically compared, one study that compared eight different nerves at 11 sites in participants aged between 21 and 80 found that the vagal nerve (sampled at the carotid bifurcation) showed no significant correlation between its cross-sectional area and age of the subject [73]. However, the state of the somatosensory and efferent autonomic neural systems in the late stages of dementia remains to be fully elucidated, and considerable work exploring the optimum taVNS stimulation parameters (current, pulse width, pulse frequency), taVNS session duration (e.g., 15 min) and chronic paradigm (e.g., once daily/during periods with accumulated attacks) is required. It is worth noting, regarding combat veterans, that Lamb et al. [74] conducted a pilot intervention study in 22 combat veterans with PTSD, concluding that taVNS positively affected the systems underlying emotional dysregulation and improved their emotional state. Thus, a thorough investigation into the autonomic activity time-wise associated with agitation in AD and the chance to influence a possible sympathetic/parasympathetic imbalance deserve greater scientific attention.

## 7. Conclusions

In the late stages of dementia, an autonomic neural imbalance seems to occur in a temporal context with transient episodes of agitated behavior and the use of transcutaneous vagal stimulations, might be a paradigm shift in the prevention and treatment of agitation in dementia diseases, including Alzheimer’s disease.
